# Hæmorrhagic Malarial Fever

**Published:** 1895-09

**Authors:** Isaac J. Jones

**Affiliations:** Austin, Texas


### TEXAS MEDICAL JOURNAL.

ESTABLISHED JULY, 1885.

PUBLISHED MONTHLY.—SUBSCRIPTION $2.00 A YEAR.

VOL. XI. AUSTIN, SEPTEMBER, 1895. NO. 3.

### Original Contributions.

For Texas Medical Journal.

### HÆMORRHAGIC MALARIAL FEVER.

BY ISAAC J. JONES, M. D., AUSTIN, TEXAS.

Read at the Austin District Medical Society.

I FEAR that a paper on the above subject will elicit but little interest from this body, the disease not being prevalent in this section, but in an unguarded moment I divulged the fact that I had the paper already prepared when I left Mississippi, and had never been delivered of it; when our secretary, in his official zeal for an essay, put me on the program.

This disease, which has been known in various localities under the synonyms of malarial hæmaturia, hæmorrhagic malarial fever, black jaundice, yellow chills, upland yellow fever, etc., appears to be indigenous to the southern States of America, and to be of recent origin.

It is true that Berranger, Ferrand and other French writers of the West Indies, and various writers of South America describe hemorrhage occurring from the kidneys during the course of malarial fevers, but this can not be the disease under discussion, for it *never* occurs after, or during the course of a remittent attack, but seems to be always connected with the remittent disease. British writers also speak of the same condition as occurring in Africa and British India, but in such a desultory and careless manner as to make it certain that they were not describing the typical and grave disease under consideration. This disease is not to be confounded with the simple hæmaturia, paroxysmal hæmoglobinuria, or the hæmaturia of the tropics, caused by the Bilharzin hæmatobia.

The disease is widely disseminated in the Southern States, but is not always co-existent with other forms of malaria, I having known a number of localities where intermittents were quite prevalent, and this disease quite unknown. Neither is it absolutely confined to malarial localities, according to some writers cases having occurred in localities immune from all other malarial manifestations.

The first accounts of the disease date back to 1840, and it was never typically described by any writer until 1868. Hence, I take it for granted that it is either of recent origin or that it has become vastly more prevalent in recent years. It has, at all events, appeared in many localities ,and become quite prevalent where previously quite unknown.

For instance, in Bolivar county, Miss., which is a very malarial locality, the first case occurred in 1860; since which time there have occurred hundreds of cases, many of which have proved fatal.

To Dr. T. C. Osborn, of Greensboro, Ala.,* belongs the honor of having first given a description of the typical disease (*N. O. Med. and Surg. Journal, 1868*), though numerous references were made to it by other writers during the twenty previous years. It was soon after reported by Drs. H. C. Ghent, of Port Sullivan, Texas; R. F. Michel and J. D. Osborn, of Alabama; Green of Georgia; B. Frank Humphrey, of Texas; John B. Pease, of Mississippi, and James Jones, of New Orleans.

Hæmorragic malarial fever is an acute disease, characterized by rigors and icteric hue of the skin and all the tissues of the body; the discharge of dark colored bloody urine; intense nausea, and nervous prostration, and usually, though not invariably, with fever.

It does not appear to be self-limited, and the tendency is to death, unless checked by appropriate treatment. The duration is from a few hours to ten or twelve days. In some cases there has been observed a tendency to recur on the seventh and fourteenth day.

I have carefully analyzed over four hundred cases of the dis-

---

*Now and for many years resident of Cleburne, Texas.

ease, as reported from various localities, and from these have selected sixty-seven that fully represent its salient features. These sixty-seven cases are used in preference to the others simply because they were fully reported.

Of these sixty-seven cases thirty-nine recovered and twenty-eight died, a mortality of 48.80 per cent. In fifty-one cases the disease was ushered in by a chill; in nine without the chill; in seven cases chill not mentioned; in thirty-two cases the initial chill was the only one experienced; twelve cases had no chills; six cases more than two; seven cases no mention. Nausea and vomiting occurred in fifty-six cases; four cases no nausea and vomiting; seven cases no mention. Thirty-nine cases had fever over 101°; ten did not, eighteen not reported. All of the sixty-seven had bloody urine, with remission of this symptom in four cases, and complete intermission in four. Sixty-four are reported as jaundiced; no mention of jaundice in three cases. There were six cases with some hemorrhage from stomach, though in only one case was it compared to the black vomit of yellow fever. One case had hemorrhage from bowels and lungs.

It will be seen by this table that the average age was twenty-five. Half the entire number were between ten and thirty, three-fourths between ten and forty, showing that the disease attacks preferably those in the prime of life. Almost twice as many males as females were attacked. While the proportion of whites to blacks is twenty to one, although the blacks outnumber the whites largely in these districts.

The subjoined table gives a complete picture of the disease. A man in the prime of life, who has been having intermittents, possibly for several months, is seized with a severe chill, or more properly, rigor, for it resembles a surgical rigor more than a malarial chill. As soon as the chill passes off, or before, the patient passes a pint or more of very dark, bloody-looking urine, which, if dropped on a piece of white paper, and allowed to dry, will make a deep yellow stain. *There are no clots or bright looking blood.*

There may be fever following chill, but it is not usually high, and, indeed, many of the severer cases run their course with a subnormal temperature. A moderately high temperature is favorable. The skin soon becomes jaundiced, varying in shade from a lemon to a deep bronze. Nausea of the most intense character is soon established, frequently accompanied by projectile vomiting. Thirst is intense. The bowels are constipated, and the stools, whether procured by mercurials or other means, are dark green and tarry. The fluids are all tinctured with bile. Many have reported that when blisters were resorted to, the vesicles filled with blood. I am persuaded that this is a mistake, as I have invariably blistered my patients and have found that the serum was of a brownish color from bile, but have seen no blood. In fact, there is no general tendency to hæmorrhage. The patient may be delirious; if so, it will be low muttering or wild excitement. The skin is very dry and hard. The urine is loaded with the material of disintegrated red blood corpuscles, epithelium from the kidney, mucus, bile, some fresh blood and albumen usually in large quantity. If the attack is of severe type, the secretion of urine begins to diminish, and unless the flow can be stimulated, will soon be entirely suppressed. Any considerable suppression means death. Of the twenty-seven cases terminated, twenty-one cases of suppression occurred. Of these eighteen died; the suppression in the other three was reported to be "slight." The patient usually has but one chill and that is a the beginning, but this is not invariable, some cases having periodical chills, with exacerbations and remissions of all the symptoms. These cases usually terminate favorably. Rigors occur at frequent intervals in severe cases, accompanied by suppression of the urine. There is but little pain complained of, and that is referred to the lumbar region. A few cases have severe colic during convalescence. The case may terminate in death or recovery in a few hours, or may continue for ten or twelve days. If an unfavorable termination can be warded off until the seventh day is passed, recovery is the rule.

The diagnosis needs no comment. It is impossible to mistake the disease.

Post mortem, the brain is jaundiced, otherwise normal; thoracic organs the same. Omentum, and its fat, of a saffron hue. Stomach full of dark greenish bile; its mucous membrane swollen and ingested. Intestines normal, except jaundice; spleen pulpy and baggy, and from three to four times its normal weight; liver slightly enlarged, soft and pulpy, chocolate color. The gall bladder filled with dark, inspissated bile. The smallest quantity of this bile will stain a whole basin of water a dark saffron hue. Kidneys enlarged, pale reddish, internally dark green. Its tubuli and malpighian tufts, choked with corpuscular debris and waste products. In severe cases these rupture giving rise to hemorrhage into the stroma of the kidneys.

In an investigation by the Committee of the Tri-State Medical Society of Alabama, Tennessee and Mississippi, acute nephritis was found in all fatal cases, a result which I have verified clinically and post mortem. The blood is very dark and does not coagulate readily. Deficient in red-blood corpuscles, and filled with the debris of the disintegrating red corpuscles and their chemical constituents, including the bile acids.

It is a mooted question whether the jaundice, which is such a constant symptom, is of hæmatogenous or hepatogenous origin. Tyson, Hare and Beranger-Ferrand favor the former, and Joseph Jones the latter view. I am inclined to think that any one who is familiar with the disease, clinically, and observes the flood of bile pouring from the kidneys and intestines, staining every tissue of the body, and even in some cases escaping through the pores of the skin, will not believe that it is all derived from the broken down blood discs. The breaking down of the corpuscles occurs in the circulation and not in the bladder, as conclusively demonstrated by Sternberg and Martin. The keystone to the pathological arch will readily be seen to be in the question, what causes this destruction of the red-blood corpuscles? I will here offer a theory which I believe is advanced for the first time:

According to Drs. R. F. Michel, Joseph Jones, and the Committee of the Tri-State Medical Society above referred to, the gall bladder, post mortem, is completely filled with bile so inspissated and thickened as to resemble a pear-shaped mass, obviously obstructing the hepatic duct. Now, Harley and others have demonstrated that the bile acids will, when thrown into the circulation, destroy the red-blood corpuscles, and by injecting them into the lower animals, have produced an hæmaturic discharge precisely similar to this disease, and followed usually by the death of the animal. Now, if the downward flow of the bile, through the hepatic duct, be obstructed, it will of necessity enter the circulation and produce the effect Harley describes. Add to this the bile that is liberated from each corpuscle, as it is destroyed, and you have a cumulative cause sufficient, in my opinion, to produce all the train of symptoms constituting the disease.

One more curious clinical fact, and I am done with this branch of the subject. It almost invariably occurs that at the end of the first chill the patient voids a pint or more of bloody urine. This amount of urine could not possibly have been secreted in this short time, hence the chill could not have caused the bloody urine; and as I have remarked above, the chill more nearly resembles a surgical rigor than the cold stage of an intermittent, hence it is my belief that the *bloody urine causes the chill or rigor.*

As to treatment, I will say but little. Quinine has been regarded as specific; but a glance at the accompanying table shows that it is equally powerless to prevent or arrest the disease. In fact, owing to the hyperæmia and imflammation of the kidneys, it is clearly contraindicated. It is also contraindicated, on account of its well known effect of lessening the oxygen-carrying power of the red-blood corpuscles. As these are the point of attack in this disease, and are so deficient in number, nothing should be done to further impair their efficiency. Indeed, Hare, Kitesato, Martin, of Mississippi, and many others, report cases where quinine has certainly caused the disease, or at least precipitated it, and I have seen it produce a relapse in cases that were convalescent. Fortunately for the patients treated with this drug, their stomachs are usually so rebellious that it is impossible to cinchonize them usually. It will be seen in the subjoined table of cases that of the sixty-seven cases tabulated, no cases died where purgatives were given and acted on the first day. Purgatives were given with effect in forty-three cases; of these only ten died, a mortality of 23.3 per cent. Given without effect in six cases, with five deaths; mortality, 83 per cent. Purgatives were not given in eleven cases, with eight deaths; mortality, 72.7 per cent. Purgatives were given—effects not stated—in three cases, with two deaths; mortality, 66⅔ per cent. Purgatives not reported on in six cases, with four deaths; mortality, 80 per cent. Hence, it will be seen that purgation is highly beneficial. This can be best secured by small doses (one or two grains) of *English* calomel every hour. This should be aided by copious enemas of normal saline solution (Dr. J. B. Pease, Miss.), repeated at least every two or three hours. This, besides aiding the action of the mercurial, stimulates the failing heart by replacing the liquor sanguinis that is being constantly lost, and encourages free diaphoresis which materially relieves the overworked kidneys. Strychina should be given hypodermically from the outset, and in doses sufficient to produce its physiological effect. Dr. J. J. Onendorf, of Sharkey county, Mississippi, assures me that he has given it in doses of ¼ gr. hypodermically every six hours with good effect. When kidneys are failing, give turpentine freely. I have always used a cantharidal blister over stomach and liver, and believe that it relieves the distressing nausea, and by relieving the congestion of the liver, aids in restoring its normal action. Theoretically jaborandi, or its active principle, should be useful in relieving the overworked kidneys, but should be given guardedly. The disease being peculiarly adynamic, supportive measures are of the utmost importance; but owing to the unfortunate condition of the stomach, but little food can be given. Beef tea, highly seasoned with red pepper, being the most useful in my experience. As the patient convalesces, perfect quietude must be enforced, as I have known a number of patients to die suddenly on slight exertion, after convalescence was thoroughly established.

